# Neuronal plasticity and fast antidepressant response

**DOI:** 10.1192/j.eurpsy.2023.68

**Published:** 2023-07-19

**Authors:** E. Castrén

**Affiliations:** Neuroscience Center / HiLIFE, University of Helsinki, Helsinki, Finland

## Abstract

**Abstract:**

Neuronal plasticity has for a long time been considered important for the recovery from depression and for the antidepressant drug action, but how the drug action is translated to plasticity has remained unclear. Brain-derived neurotrophic factor (BDNF) and its receptor TRKB are critical regulators of neuronal plasticity and have been implicated in the antidepressant action. We have recently found that many, if not all, different antidepressants, including serotonin selective SSRIs, tricyclic as well as fast-acting ketamine, directly bind to TRKB, thereby promoting TRKB translocation to synaptic membranes, which increases BDNF signaling. We have previously shown that antidepressant treatment induces a juvenile-like state of activity in the cortex that facilitates beneficial rewiring of abnormal networks. It is important to note that enhanced plasticity does not necessarily promote recovery, but may also be maladaptive if the environment is adverse. Our findings open a new framework for the antidepressant action and for treatment of depression: antidepressants directly bind to TRKB and allosterically promote BDNF signaling, thereby inducing a state of plasticity that allows re-wiring of abnormal networks for better functionality, when optimal supportive therapy is provided at the time of enhanced plasticity.

**Disclosure of Interest:**

E. Castrén Speakers bureau of: Janssen-Cilag

